# Prevalence of methicillin-resistant *Staphylococcus aureus* skin and nasal carriage isolates from bovines and its antibiogram

**DOI:** 10.14202/vetworld.2017.593-597

**Published:** 2017-06-04

**Authors:** Alok Kumar, Purushottam Kaushik, Anjay, Pankaj Kumar, Manoj Kumar

**Affiliations:** 1 Department of Veterinary Public Health & Epidemiology, Bihar Veterinary College, Patna, Bihar, India; 2 Department of Veterinary Microbiology, Bihar Veterinary College, Patna, Bihar, India

**Keywords:** antibiogram, bovine, *mec* A gene, methicillin resistant *Staphylococcus aureus*

## Abstract

**Aim::**

This study was conducted to determine the prevalence of methicillin-resistant *Staphylococcus aureus* (MRSA) in cattle and buffalo and to study their antibiotic resistance pattern.

**Materials and Methods::**

A total of 136 samples (skin and nasal swab) from cattle and buffalo were collected. MRSA was identified by conventional bacterial culture techniques which were further confirmed by amplification of *S. aureus*-specific 16S rRNA by polymerase chain reaction (PCR). The isolates were further analyzed for the presence of *mec* A gene by PCR. The antimicrobial susceptibility profiling was performed by disc diffusion method.

**Results::**

The prevalence of MRSA in the current study was 28.57% and 34.28% in cattle nasal and skin swab, respectively, with an overall prevalence of 31.43% MRSA among cattle. Buffalo nasal and skin sample showed MRSA prevalence of 54.55% and 39.4%, respectively, with 46.9% overall prevalence. PCR could detect *mec* A gene in 36.4% and 58% MRSA isolates from cattle and buffalo, respectively. Antimicrobial susceptibility test found MRSA resistant to penicillin and oxytetracycline (88% each), cefoxitin (75%), cotrimoxazole (62%), and amoxyclav (50%). 100% sensitivity was observed against ciprofloxacin, amikacin, chloramphenicol, and gentamicin. Three (16.7%) MRSA isolates from buffalo were found resistant to vancomycin.

**Conclusion::**

Cattle and buffalo were identified as a potential carrier of MRSA in Bihar (India). The isolation of vancomycin-resistant *S. aureus* (VRSA) in the current study indicates the emergence of VRSA in animal population which may be transmitted to the human beings working in close contact to the animals.

## Introduction

*Staphylococcus aureus* is an opportunistic pathogen of human and animal [1] which usually colonizes anterior nares and causes infection in immunocompromised patients [2]. Alexander Ogston in 1880 first isolated *S. aureus* and first isolation of penicillin-resistant *S. aureus* was made in 1942 from clinical cases [3]. It was later on circumvented by the introduction of methicillin and the first methicillin resistant *S. aureus* (MRSA) appeared in the year 1961 which later on became a serious nosocomial infection worldwide [4]. The organism has developed resistance to methicillin by integration of a 21-67 kbp mobile genetic element, termed as staphylococcal cassette chromosome mec [5] into their genome which harbors the methicillin resistance (*mec*A) gene and other antibiotic resistance determinants [6,7], leaving vancomycin as the drug of last choice to treat the MRSA infections. This gene encodes for a penicillin-binding protein 2 (PBP2a) expressed in the bacterial cell wall and has a low affinity for β-lactam antibiotics. Thus, this group of antibiotics becomes insensitive to bacteria expressing mecA gene.

Data regarding the prevalence of MRSA in India have been reported from various regions [8-11]. However, no systematic study is available on the prevalence and antimicrobial resistance pattern of MRSA, in Bihar.

Hence, this study was planned for isolation, identification and molecular detection of methicillin resistance in staphylococci and their antibiotic resistance pattern from bovines.

## Materials and Methods

### Collection of sample

Skin and nasal swab samples (136) from apparently healthy cattle and buffalo were collected from Institutional Livestock Farm Complex, Bihar Veterinary College (BVC), Patna, and from the patients coming to teaching veterinary clinical complex of BVC, Patna. The sample comprised skin and anterior nostril swab of cattle (35 each) and buffalo (33 each). The skin samples were collected from the axillary region of the animals. The samples were inoculated in tryptone soya broth (TSB) with 10% sodium chloride salt (TSB-S) (Hi-media) and incubated at 37°C for 24 h. The samples which showed turbidity in TSB-S broth were streaked on mannitol salt agar (Hi-media) with 6 mg/L oxacillin (O-MS agar) and incubated for 24 h at 37°C. Bacterial growth with mannitol fermentation was observed for the presence of round-shaped, typical golden, yellow, or pale color colonies of oxacillin-resistant staphylococci. The presumptive isolates were further confirmed by Gram’s-staining (Hi-media) and catalase test [12]. The test was performed with a loopful culture of isolates mixed with a drop of 3% aqueous solution of hydrogen peroxide observed for the production of gas bubbles by catalase-positive isolates. The oxacillin resistant, mannitol fermenter, Gram-positive cocci in bunch showing positive catalase activity were considered as MRSA isolates. Further these positive isolates were confirmed by *S. aureus*-specific polymerase chain reaction (PCR) targeting 16S rRNA using the primer sequence as F-5’ GTAGGTGGCCAAGCGTTATCC 3’ and R-5’ CGCACATCAGCGTCAG 3’ [13]. The PCR was performed with 1X PCR buffer (pH-8.3; 15 mM MgCl_2_), 5 mM of deoxynucleotide triphosphates (dNTPs), 20 pmol of forward and reverse primers, 1 U Taq DNA polymerase, 2 µl of DNA template per reaction and final volume was adjusted to 25 µl with nuclease free water. The cycling conditions were optimized with initial denaturation at 94°C for 5 min, followed by 35 cycles of denaturation at 94°C for 1 min, annealing at 50°C for 1 min, and extension at 72°C for 1 min with the final extension at 72°C for 10 min.

The confirmed isolates of MRSA were further screened for the presence of *mecA* gene by PCR. The PCR assay was standardized for amplification of *mec*A gene by forward (5’ GTA GAA ATG ACT GAA CGT CCG ATAA 3’) and reverse (5’ CCAATTCC ACATTGT TTCG GTC TAA 3’) primer [14] with some modifications. The PCR reaction mixture was prepared in 25 µl reaction volume each containing 2.5 µl ×10 PCR buffer (pH-8.3; 15 mM MgCl_2_), 0.5 µl of dNTP mixture (10 mM each), 2 µl (10 pmol/µl) of forward and reverse primers, 1 µl (1 unit) Taq DNA polymerase, 5 µl of bacterial lysate and final volume was adjusted with nuclease free water. The bacterial lysate was prepared by boiling and snap chilling [15] from overnight grown culture in TSB. The cycling conditions used were initial denaturation at 94°C for 5 min, followed by 32 cycles of denaturation at 94°C for 1 min, annealing at 56°C for 1 min and extension at 72°C for 1 min with the final extension phase at 72°C for 10 min.

The amplified products were analyzed by agarose gel electrophoresis using 1.5% LE agarose (Hi media, India) on tris acetate-ethylenediaminetetraacetic acid buffer containing ethidium bromide (0.5 µg/ml). Gels were visualized and photographed under gel documentation system (Biorad).

Antibiogram study of MRSA isolates was performed by disc diffusion method [16] using a group of antibiotics, *viz*., ciprofloxacin (5 µg), amoxiclav (30 µg), ofloxacin (5 µg), amikacin (30 µg), cefoxitin (30 µg), ceftriaxone (30 µg), chloramphenicol (30 µg), clindamycin (2 µg), penicillin (10 µg), gentamicin (10 µg), oxytetracycline (30 µg), tetracycline (30 µg), cotrimoxazole (25 µg), and vancomycin (30 µg). The interpretation of results was made according to CLSI guidelines [9].

## Results

A total of 136 nasal and skin swab samples were processed in this study. The prevalence of MRSA, out of 136 nasal and skin swab samples studied, is shown in Table-1. 22 and 31 MRSA isolates were collected from cattle and buffalo respectively, based on biochemical confirmation and amplification of *S. aureus*-specific 16S rRNA (228 bp) gene (Table-1). The prevalence of MRSA was 28.57% and 34.28% in cattle nasal and skin swabs, respectively, with an overall prevalence of 31.43% among cattle, whereas buffalo nasal and skin showed 54.55% and 39.4%, respectively, with an overall prevalence of 46.9% MRSA among buffalo (Table-1).

**Table-1:** Prevalence of MRSA among cattle and buffalo.

Sample type	n=136	% MRSA detected by mannitol fermentation and 16S rRNA amplification (n)	% *mecA* positive MRSA (n)
Cattle			
Nose swab	35	28.57 (10)	50.00 (05)
Skin swab	35	34.28 (12)	25.00 (03)
Sub total	70	31.43 (22)	36.36 (08)
Buffalo			
Nose swab	33	54.55 (18)	50.00 (09)
Skin swab	33	39.39 (13)	69.23 (09)
Sub total	66	46.97 (31)	58.06 (18)

N: Total number of samples; n: number of samples in each column. MRSA=Methicillin resistant *Staphylococcus aureus*

**Figure-1:**
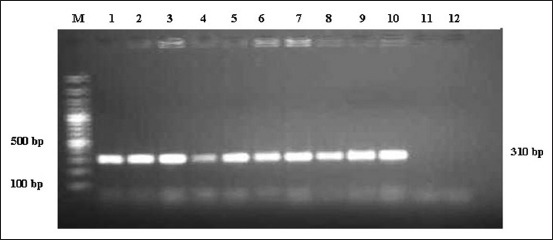
Polymerase chain reaction amplification of *mec*A gene of methicillin resistant *Staphylococcus aureus*. M: Gene ruler 100 bp plus DNA ladder, L1: Positive control, L2-L10: Positive amplicons of methicillin resistant *Staphylococcus aureus*, L11: Isolate with no amplicon, L12: Negative control.

PCR amplification of the *mec*A gene has been used for specific and rapid identification of MRSA among the oxacillin-resistant *S. aureus* isolates. The test detected *mec*A gene (Figure-1) in 58.06% MRSA isolates from buffalo, whereas only 36.4% of MRSA isolates from cattle were found positive for *mec*A gene. The frequency of detection of mecA gene in cattle nostril and skin swab was 50% and 25%, respectively, wherein it was 50% and 69.23% in buffalo nostril and skin swab, respectively (Table-1).

**Figure-2:**
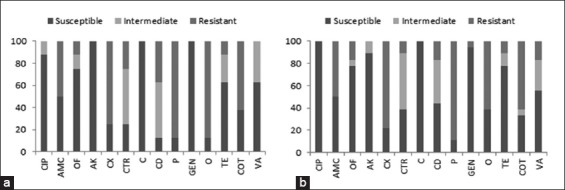
Antimicrobial susceptibility test result for methicillin resistant *Staphylococcus aureus* from cattle (a) and buffalo (b). CIP: Ciprofloxacin, AMC: Amoxiclav, OF: Ofloxacin, AK: Amikacin, CX: Cefoxitin, CTR: Ceftriaxone, C: Chloramphenicol, CD: Clindamycin, P: Penicillin, GEN: Gentamicin, O: Oxytetracycline, TE: Tetracycline, COT: Cotrimoxazole and VA: Vancomycin.

Antimicrobial susceptibility test was performed for all the 53 MRSA isolates of cattle and buffalo origin. It revealed that 88% MRSA isolates of cattle origin, having *mec*A gene were resistant to penicillin and oxytetracycline, whereas 75% were resistant to cefoxitin followed by cotrimoxazole (62%) and amoxyclav (50%) whereas no resistance was observed against ciprofloxacin, amikacin, chloramphenicol, gentamicin, and vancomycin (Figure-2a). Similar pattern of antimicrobial resistance for penicillin, cotrimoxazole, cefoxitin, ciprofloxacin, amikacin and chloramphenicol was observed in *mec*A positive isolates of buffalo origin. The finding also revealed resistance against vancomycin, in 16.7% of MRSA isolates from buffalo (Figure-2b).

## Discussion

The findings of this study revealed that prevalence of MRSA in buffalo (46.9%) was higher in comparison to cattle (31.43%) which corroborate with earlier findings on isolation of MRSA from nasal or skin samples of cattle [17,18]. However, no reports are available on isolation of MRSA from buffalo nasal and skin samples. Although low prevalence (0.7%) of MRSA harboring in nose and skin of pigs have been reported from Thailand [19]. The difference in the sample size and geographical variations may be the causes of this discrepancy in the prevalence of MRSA. The presence of *mec*A gene which encodes a modified penicillin-binding protein (PBP), i.e., PBP2a is a useful molecular marker of β-lactam resistance in *Staphylococci* [20,21]. Hence, PCR amplification of the *mec*A gene have been used in the present study for specific identification of MRSA among the oxacillin-resistant *S. aureus* isolates [22,23]. The study revealed a total 36.36% and 58.06% of MRSA isolates carrying *mec*A gene from cattle and buffalo, respectively. Similar findings of mecA gene detection in cattle nasal carriage have been reported by Alzohairy [24]. In contrast, a lower prevalence of 0.3% and 1% of MRSA has been reported in farm cattle and calves [25]. The lower percentage of *mecA* gene amplification by PCR among MRSA isolates in the current study may be due to penicillinase activity of *S. aureus* [26] or due to the presence of new *mec*A gene homologue, mecALGA251 called *mec*C gene in the isolates [27] which could be responsible for MRSA isolates.

The antibiogram study revealed resistance to penicillin and oxytetracycline (88%) in MRSA isolates of cattle, whereas 75% were resistant to cefoxitin, (62%) cotrimoxazole and (50%) amoxyclav. No resistance was observed against ciprofloxacin, amikacin, chloramphenicol, gentamicin and vancomycin. Similar pattern of antimicrobial resistance for penicillin, cotrimoxazole, cefoxitin, ciprofloxacin, amikacin, and chloramphenicol was observed in *mec*A positive isolates of buffalo origin. It revealed that 88% MRSA isolates of cattle origin, having *mec*A gene were resistant to penicillin and oxytetracycline whereas 75% were resistant to cefoxitin followed by cotrimoxazole (62%) and amoxyclav (50%) whereas no resistance was observed against ciprofloxacin, amikacin, chloramphenicol, gentamicin, and vancomycin (Figure-2a). This antimicrobial resistance pattern was found similar to that of buffalo isolates. The finding also revealed resistance against vancomycin, in 16.7% of MRSA isolates from buffalo. Kumar *et al*. [8], Vishnupriya *et al*. [9], and Chandrasekaran *et al*. [10] have the similar findings of antimicrobial resistance against a group of antibiotics in MRSA.

The current finding also revealed resistance against vancomycin, in 16.7% of MRSA isolates from buffalo which is considered as the drug of choice for treatment of MRSA infections [28]. Although a low level of resistance against vancomycin has been reported earlier [28], only a few intermediate susceptibility but no resistance was against vancomycin has been reported [11]. Hence, the isolation of vancomycin-resistant *S. aureus* (VRSA) in the current study indicates the emergence of VRSA in animal population which may have a serious implication in human infection.

## Conclusion

The present finding concludes that cattle and buffalo may act as a potential carrier of MRSA which may act as a risk factor for human’s infection who are in direct contact with live animals [29].

## Authors’ Contributions

PK was the mentor and project leader. AK and Anjay were responsible for project design and performed most of the work. PK and MK were responsible for experimental work and support. All authors read and approved the final manuscript.
